# Efficient leather spreading operations by dual-arm robotic systems

**DOI:** 10.1038/s41598-024-66904-2

**Published:** 2024-07-15

**Authors:** Yuan Huan, Gongchang Ren, Jiangong Sun, Guang Jin, Xujiang Ding, Wenhao Du

**Affiliations:** College of Mechanical and Electrical Engineering, Shaanxi University of Science and Technology, Xi’an, China

**Keywords:** Non-uniform and sheet-like soft objects, Grasping and unfolding techniques, Optimal grasping points, YOLOV8 improvement, Grasping posture optimization, Engineering, Mechanical engineering

## Abstract

To achieve precise grasping and spreading of irregular sheet-like soft objects (such as leather) by robots, this study addresses several challenges, including the irregularity of leather edges and the ambiguity of feature recognition points. To tackle these issues, this paper proposes an innovative method that involves alternately grasping the lowest point twice and using planar techniques to effectively spread the leather. We improved the YOLOV8 algorithm by incorporating the BIFPN network structure and the WIOU loss function, and trained a dedicated dataset for the lowest grasping points and planar grasping points, thereby achieving high-precision recognition. Additionally, we determined the optimal posture for grasping the lowest point and constructed an experimental platform, successfully conducting multiple rounds of leather grasping and spreading experiments with a success rate of 72%. Through an in-depth analysis of the failed experiments, this study reveals the limitations of the current methods and provides valuable guidance for future research.

## Introduction

With rapid economic development, leather has been extensively employed due to its durability and superior appearance, leading to an increasing demand for leather products. However, the production process of leather is intricate, involving numerous stages, which make the manufacturing process laborious. Despite decades of development in leather processing equipment, which has led to automation or semi-automation in various stages, manual assistance remains indispensable, especially in processes that require the spreading of leather from a disordered piled state to a flat configuration. The manual intervention in the production process poses significant challenges to further enhancing production efficiency. With technological advancements, replacing manual labor by robotic technology and improving production efficiency has emerged as an effective strategy, particularly in the labor-intensive and harsh working conditions of the leather manufacturing industry. Because leather are irregularly shapedand soft sheet materials, which have the characteristics of large flat area, small thinness, and susceptible to deformation, spreading such materials by robots presents considerable challenges.

Numerous studies have focused on robotic manipulation of garments or towels. In Reference^1^, grasping cloth by a robotic arm at arbitrary points, with cloth hanging up naturally under gravity, then machine vision is used to identify key points in the clothing's contour for dual-arm grasping and unfolding. Reference^2^ discusses the expansion of grasping points' distance after repeated grasping of the lowest points on a garment by two robotic arms to achieve spreading. Reference^3^ employs a PR2 robot for folding and identifying garmentsby matching the observed shapes with known templates to determine grasping points primarily. Reference^4^ involves 3D reconstruction of garment features for posture recognition through database matching. Reference^5^ achieves towel folding through visual identification of towel edges and corners in conjunction with a designed dual-end effector. Reference^6^ utilizes a depth sensor to detect the contours and wrinkles of clothes laid on a table to calculate grasping points and unfolding directions. Reference^7^ unfolds towels by locating their edges using tactile and visual sensors. Research related to the grasping and spreading of garments or towels can be summarized as inferring suitable grasping points through methods such as visual imaging or deep learning, followed by the execution of unfolding or spreading steps^8–19^.

Research on the grasping and spreading of sheet-like soft materials has focused on garments or towels primarily. Since garments or towels are man-made items, which have distinct features such as regular edges, corners, or conspicuous characteristic points, making spreading tasks feasible through template matching. However, influencing by animal individual differences and the skinning process, leather has irregular edges lacks the distinct feature patterns found in regular sheet-like soft materials such as clothes and towels, which makes it difficult to identify grasping points by locating specific features. Additionally, the leather processing involves various chemical or physical treatments, incorporating numerous chemical agents, resulting in a surface covered in stains, which makes it impractical to rely on direct-contact sensors for assisting in the grasping and spreading of leather. Moreover, available literatures focus on identifying grasping points and designing end effectors primarily, but the grasping posture is equally crucial for improving the success rate of operations.

Therefore, on the basis of studying various methods of grasping and spreading clothes and towels, combining the irregular edges, surface stains, and unclear characteristic recognition points, this paper develops grasping and spreading methods suitable for irregular shaped soft sheet material such as leather. Utilizing deep learning for identifying grasping points and determining the optimal grasping posture through experiments, this study addresses the challenge of using robots for grasping and spreading leather in the production process effectively.

## Study on the grasping and spreading scheme

**Figure 1:**
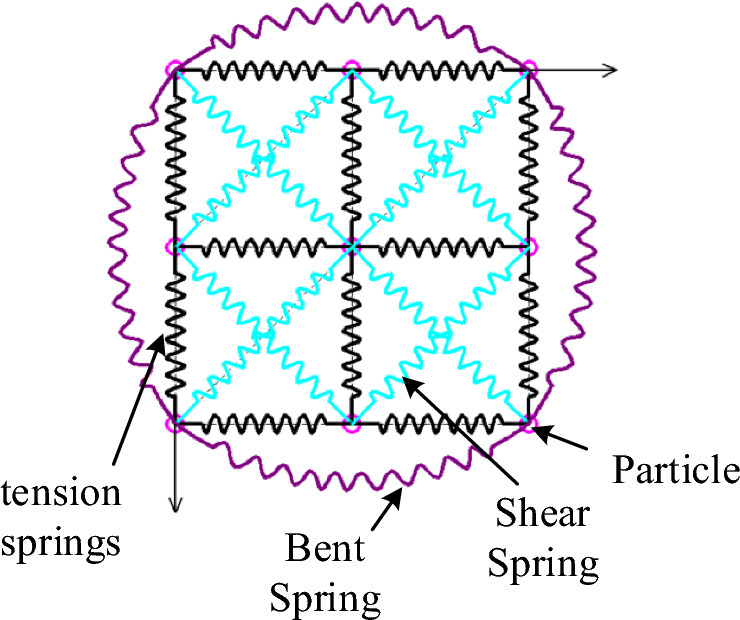
Proton-spring model.

**Figure 2:**
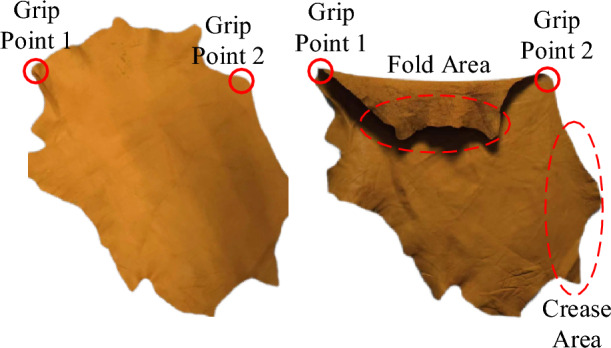
Deformation diagram of the leather grasping process.

To derive a feasible method for the grasping and spreading of leather, the forces acting on the leather during the grasping process are analyzed through the mass-spring model. As illustrated in Fig. 1, the mass-spring model represents the deformation process of leather through the forces exerted on masses by tension springs, shear springs, and bending springs^20,21^. In the process of grasping and spreading sheet-like soft materials, the primary forces acting on a mass include its own gravity, damping force, internal spring tension, and external force (exerted by the robotic end effector interaction with the leather). When all masses achieve equilibrium on a plane parallel to the gravitational field under the influence of various forces, the sheet-like soft material is considered to be in a spread state, similar to a towel being grasped at two adjacent corner points. However, when the number of grasping points is limited, for irregularly edged sheet-like soft materials, the masses located outside the grasping points near the edges struggle to achieve balance on a plane parallel to the gravitational field due to gravity, resulting in local wrinkles and folds. As shown in Fig. 2, when leather is lifted by grasping at two points, areas of folding and wrinkling may appear outside the two grasping points due to gravity, especially for leather with a large span. The analysis using the mass-spring model also indicates that sheet-like soft materials can’t withstand forces perpendicular to their surface, making it difficult for all masses of an irregularly edged sheet-like soft material to achieve balance on a plane parallel to the gravitational field through a limited number of grasping points. Taking the characteristics of leather and the actual conditions of processing into account, it is necessary to utilize a plane to achieve the spreading of leather. Moreover, due to the lack of distinct feature points on leather, the lowest points must be chosen as feature recognition points. After extensive experimentation, the LTPP (Lowest-Two-Point-Plane) method was proposed for the grasping and spreading of leather. This method achieves effective grasping and spreading of irregular sheet-like soft materials by alternately grasping the two lowest points of the material and utilizing a plane for assistance.

**Figure 3:**
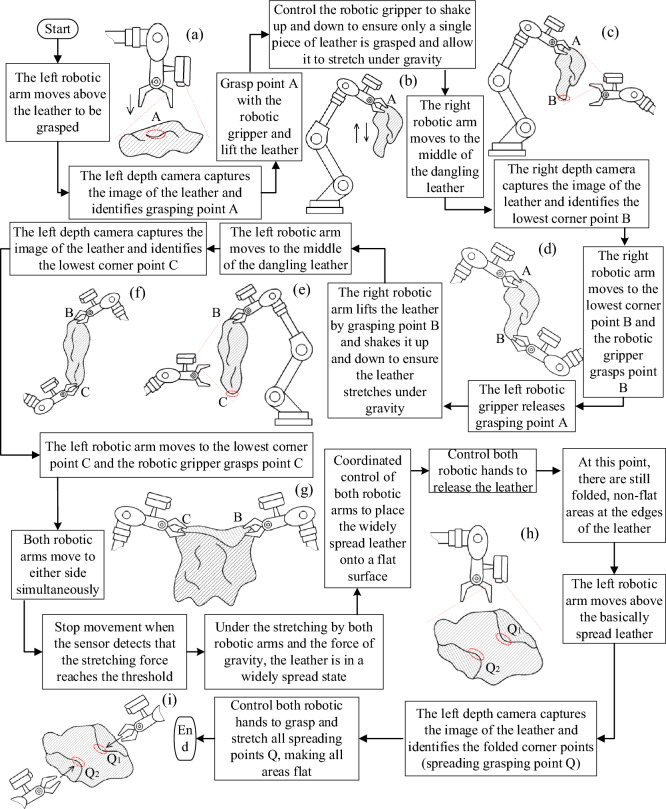
Flowchart of the leather grasping and spreading method.

Considering the specific operational environment of the leather production process, an eye-in-hand visual system layout was selected. An independent dual-arm robot was deployed on both sides of the workstation to provide comprehensive visual coverage and operational range for the robotic system within the work area. The proposed process for using dual robotic arms to grasp and spread leather is illustrated in Fig. 3. Initially, the left robotic hand performs a preliminary grasp on the leather in a coiled and stacked state. This grasp does not require specific feature points and can be performed at any point where the leather can be securely held. Through the up-and-down shaking motion of the robotic arm, the leather is encouraged to separate from its original coiled and stacked state, achieving preliminary relaxation and disentanglement. Subsequently, machine vision is used to identify the lowest corner point. After the right robotic hand grasps the lowest point, the left robotic hand releases it, and the right hand performs an up-and-down shaking motion to further relax the leather. Machine vision is then used again to identify the lowest grasping point, and the left robotic hand grasps this point. Both robotic hands then simultaneously stretch the leather to the sides. At this stage, the leather unfolds extensively under the influence of gravity. The two robotic arms then collaborate to place the extensively unfolded leather on a flat surface. Machine vision is used to identify any remaining unspread areas and flat spreading points, and the robotic hands grasp these points to fully spread the leather over all areas.

## Grasping point identification and localization algorithm

### Enhanced YOLOV8 algorithm

Among various target detection algorithms, the YOLO (You Only Look Once) series stands out due to its rapid processing and high accuracy, making it extensively adopted across diverse domains^22,23^. As the latest iteration within the YOLO series, YOLOV8 achieves an optimal balance between speed and accuracy. It introduces a state-of-the-art (SOTA) model that enhances detection precision and efficiency while reducing network parameters. However, the existing YOLOV8 algorithm struggles to extract and integrate the characteristic information of leather for grasping point detection effectively. Therefore, it is necessary to refine the current YOLOV8 model and improve its generalization capability significantly.

**Figure 4:**
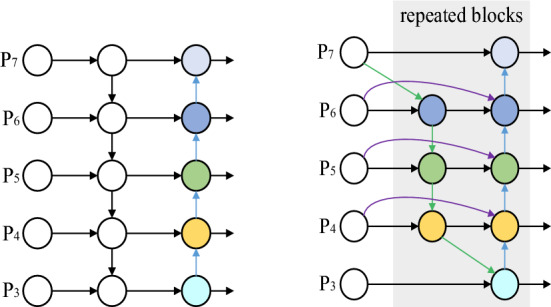
Structure of the PANet and BiFPN network models.

**Figure 5:**
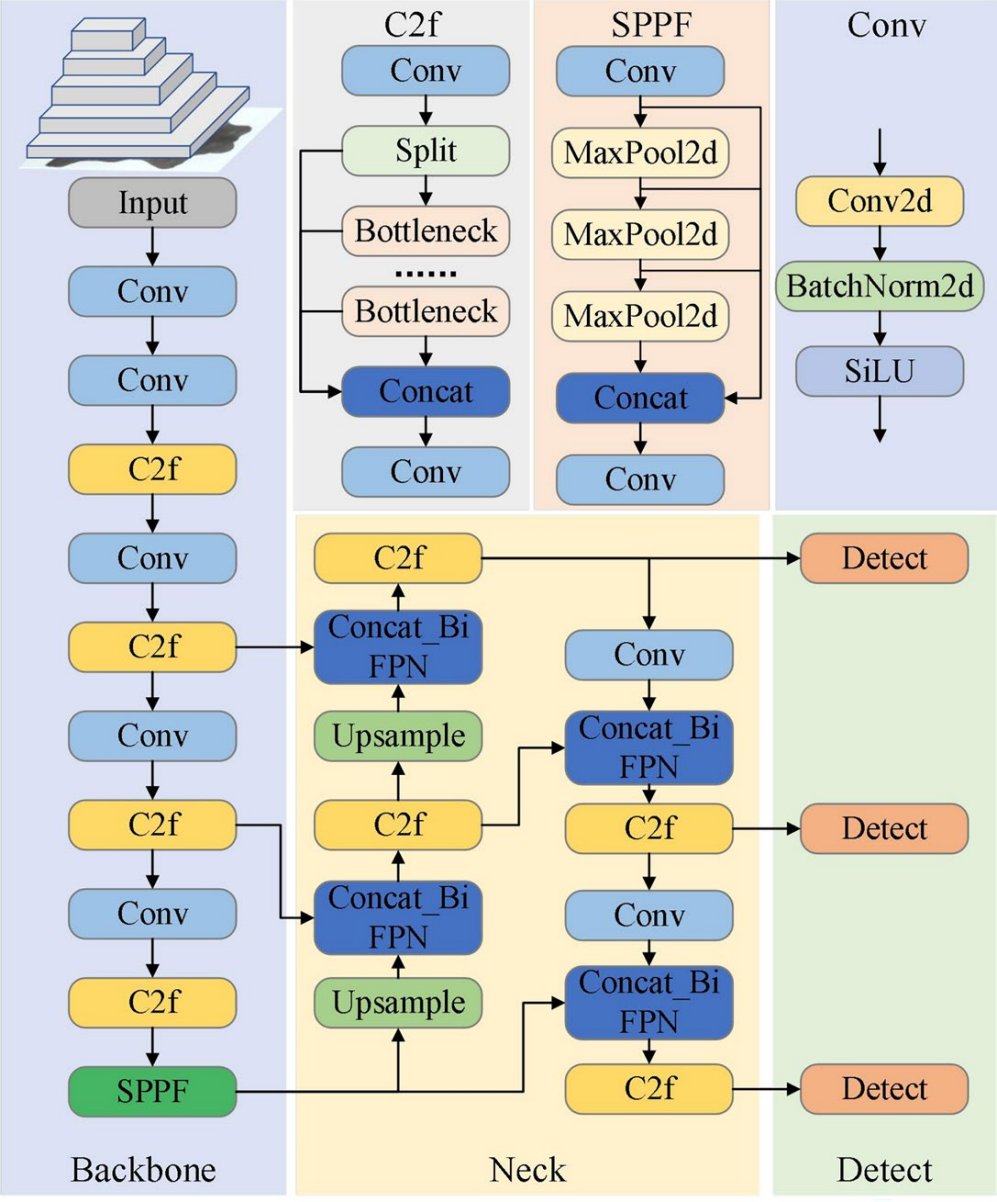
Improved YOLOv8 model network structure.

The Bi-directional Feature Pyramid Network (BIFPN), a feature fusion network for target detection tasks, employs a bi-directional feature pyramid structure, enhances feature expression and detection performance by effectively extracting and integrating multi-scale information in images through learned specific weights. This approach the model structure of PANet and BIFPN are illustrated in Fig. 4. BIFPN, different from PANet, eliminates nodes with a single input edge and introduces bi-directional connections between different levels in the middle layers. This structure ensures each feature layer encompasses feature maps from various scales, facilitating the effective integration of higher-level features and improving the network's ability to process targets of differing scales, thereby enhancing detection precision. For instance, the P6 feature fusion in Fig. 5 can be represented by a weighted bi-directional pyramid network structure as follows:

$$ \begin{array}{*{20}r} \hfill {P_{6}^{td} } & \hfill { = Conv\left( {\frac{{w_{1} \cdot P_{6}^{in} + w_{2} \cdot Resize\left( {P_{7}^{in} } \right)}}{{w_{1} + w_{2} +\epsilon }}} \right)} \\ \hfill {P_{6}^{out} } & \hfill { = Conv\left( {\frac{{w_{1}{\prime} \cdot P_{6}^{in} + w_{2}{\prime} \cdot P_{6}^{td} + w_{3}{\prime} \cdot Resize\left( {P_{5}^{out} } \right)}}{{w_{1}{\prime} + w_{2}{\prime} + w_{3}{\prime} + \epsilon}}} \right)} \\ \end{array} $$

where *Resize* denotes down-sampling or up-sampling operations, *Conv* represents convolution operations, *w* denotes the weight of each layer, *P* represents the features of the corresponding layer, and *e* denotes the learning rate.

This paper integrates the BIFPN feature enhancement module into YOLOV8, refining the Neck part of the YOLOV8 model, which boost detection accuracy significantly by multi-level feature pyramid and bi-directional information transfer.

The Weighted Intersection over Union (WIOU) is a loss function for target detection that addresses the issue of requiring high-quality training data in traditional bounding box loss functions. The method measures the overlap between predicted and true boxes by computing the Intersection over Union (IoU) and then weighting it with the minimum weights of parts in both boxes. This approach enhances target detection accuracy by emphasizing samples with smaller IoU valueswhich are harder to detect. Given the harsh conditions of leather processing, training data often include low-quality images. Replacing the loss function in YOLOV8 with WIOU prevents a decline in the model's generalization ability and addresses the imbalance in leather sample quality effectively. WIOU has three versions, for WIOUV1, dual-layer attention mechanism by introducing a distance attention built through distance metrics. versions V2 and V3 are obtained by adding gradient enhancement to the Focus mechanism in WIOUV1. The function definition for WIOUV1 is as follows:

$$\begin{array}{cc}& {\mathcal{L}}_{WIoUv1}={\mathcal{R}}_{WIoU}{\mathcal{L}}_{IoU}\\ & {\mathcal{R}}_{WIoU}=\text{exp}\left(\frac{{\left(x-{x}_{gt}\right)}^{2}+{\left(y-{y}_{gt}\right)}^{2}}{{\left({W}_{g}^{2}+{H}_{g}^{2}\right)}^{*}}\right)\end{array}$$

Here, *Wg* and *Hg* represent the width and height of the minimum bounding box, and *RWIoU* denotes the normalized distance between the center points of the predicted and true boxes.

By incorporating the BIFPN network structure and WIOU loss function, the traditional YOLOV8 algorithm is enhanced, which addresses the challenges of inadequate feature information extraction and integration for leather grasping point detection, as well as the issue of model generalization due to imbalanced leather sample quality. The model network structure of improved YOLOV8 is depicted in Fig. 5.

### Principle of grasping point localization

YOLOV8, utilizing anchorless detection, is capable of predicting the center of the target object directly, which determines the coordinates of the bounding box accurately and enhances object localization precision significantly by predicting the distance from the center to the edges. The improved YOLOV8 algorithm facilitates the acquisition of the grasping points' image coordinates within the leather RGB images. Precise localization of grasping points in three-dimensional space can be achieved by registering the depth information obtained from the D435i depth camera with the RGB image and conversion of pixel coordinates to world coordinates.

**Figure 6:**
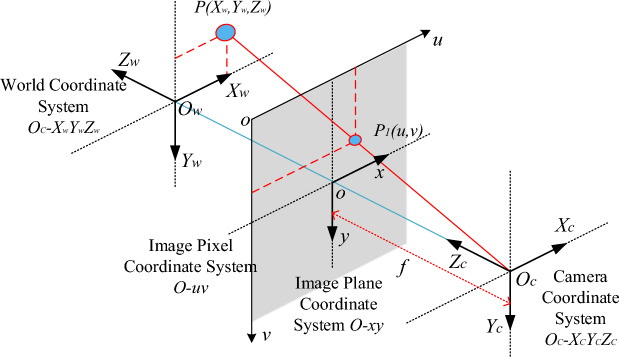
Coordinate system transformation diagram.

The transformation from pixel coordinate system to world coordinate system involves sequential conversions through pixel, image, and camera coordinate systems, as illustrated in Fig. 6. This multi-step process ensures the accurate translation of image data into spatial positioning, which is crucial for robotic interaction in a real-world environment.

The transformation from world coordinates to camera coordinates, then to image coordinates, and finally to pixel coordinates is delineated by the following relationship:

$$\begin{array}{c}{Z}_{c}\left[\begin{array}{c}u\\ {v}_{1}\end{array}\right]=\left[\begin{array}{ccc}\frac{1}{dx}& 0& {u}_{0}\\ & & \\ 0& \frac{1}{dy}& {\nu }_{0}\\ 0& 0& 1\end{array}\right]\left[\begin{array}{cccc}f& 0& 0& 0\\ 0& f& 0& 0\\ 0& 0& 1& 0\end{array}\right]\left[\begin{array}{cc}R& T\\ \overrightarrow{0}& 1\end{array}\right]\left[\begin{array}{c}{X}_{w}\\ {Y}_{w}\\ {Z}_{w}\\ 1\end{array}\right]=\left[\begin{array}{cccc}{f}_{x}& 0& {u}_{0}& 0\\ 0& {f}_{y}& {\nu }_{0}& 0\\ 0& 0& 1& 0\end{array}\right]\left[\begin{array}{cc}R& T\\ \overrightarrow{0}& 1\\ 1& 1\end{array}\right]\left[\begin{array}{c}{X}_{w}\\ {Y}_{w}\\ {Z}_{w}\\ 1\end{array}\right]\\ \end{array}$$

where: *f* is the focal length of the camera; *dx* and *dy* are pixel sizes; *f*_*x*_*, f*_*y*_*, u*_*0*_ and ν_0_ are internal camera parameters, and *R* and *T* are external camera parameters.

The spatial coordinates of the leather grasping points can be obtained through the coordinate transformation formula after RGB and depth images registration which are captured by D435I depth camera.

## Validation of grasping point identification algorithm

### Dataset creation and preprocessing

Taking into account the cluttered environment and variable lighting conditions inherent in leather processing, the datasets for the lowest grasping points and flat spreading grasping points are compiled under different lighting and background conditions, consisting of 5000 and 1000 images respectively. The datasets is split into training and testing sets at a ratio of 7:3. The datasets is annotated by software Labelme, which categorizes the annotations into two classes: ‘leather’ and ‘point’ for the lowest grasping points and flat spreading grasping points respectively. The annotation *json* files are then converted into *txt* format for subsequent model training.

### Evaluation metrics and experimental configuration

The chosen evaluation metrics for the algorithm's performance are Precision (P), Recall (R), and mean Average Precision (mAP). Precision represents the accuracy, which is the proportion of true positive results in all positive predictions. Recall measures the ability of the model to detect all relevant instances within the dataset. Mean Average Precision (mAP) is the mean of the average precision scores for each class, providing an overall effectiveness measure of the algorithm across different classes and thresholds.

$$\begin{array}{c}\text{P}=\frac{\text{TP}}{\text{TP}+\text{FP}}\\ \text{R}=\frac{\text{TP}}{\text{TP}+\text{FN}}\\ \text{mAP}=\sum_{\text{i}=0}^{\text{N}}\frac{{\text{AP}}_{\text{i}}}{\text{N}}\end{array}$$

In the formula, *TP* (True Positives) refers to the count of correctly predicted positive samples, *FN* (False Negatives) to the number of positive samples incorrectly predicted as negative, and *FP* (False Positives) to the count of negative samples incorrectly predicted as positive.

The experiments are conducted on a system running Windows 11, equipped with an NVIDIA GeForce RTX 3060 GPU with 12 GB of VRAM and 64 GB of system memory. The algorithm model is built using the Pytorch framework, with CUDA version 11.3, and employing an Intel RealSense D435i depth camera as the visual sensor. During the training phase of the model, the training parameters are set as follows: epochs (number of iterations) at 300, batch size at 32, and learning rate at 0.01.

### Experimental results and analysis

To validate the performance of the proposed algorithm, comparative experiments are conducted using two sets of training datasets under consistent conditions regarding the number of iterations, optimizer, learning rate, batch size, and training equipment. The experiments compare the enhanced YOLOV8 with the original YOLOV8, YOLOV7, YOLOV5, and Faster R-CNN algorithms. The results of the trained algorithms are presented in Table 1. According to the experimental outcomes in Table 1, the YOLO series algorithms demonstrate a significant advantage over the Faster R-CNN in terms of speed and accuracy in detecting the lowest and flat spreading grasping points on leather. Among YOLOV5, YOLOV7, YOLOV8, and the enhanced YOLOV8, the enhanced YOLOV8 shows a notable improvement in Precision (P) and mean Average Precision (mAP). Although the Recall (R) of the enhanced YOLOV8 is lower compared to YOLOV5, its overall detection performance and speed are superior. The training results indicate that the enhanced YOLOV8 improves the identification and localization of the lowest and flat spreading grasping points on leather effectively.

**Table 1:** Comparison of model algorithms.

Algorithm model	Lowest grasping Point dataset			Spreading grasping Point dataset		
	P/%	R/%	mAP/%	P/%	R/%	mAP/%
Faster R-CNN	92.08	90.13	91.15	93.23	90.82	92.03
YOLOv5	96.35	**98.02**	99.08	97.28	**98.32**	99.01
YOLOv7	97.38	97.06	98.13	99.65	96.15	98.29
YOLOv8	97.90	97.17	98.42	99.23	97.78	98.64
Improved YOLOv8	**98.42**	97.80	**99.17**	**99.74**	98.16	**99.05**

Significant values are in bold.

**Figure 7:**
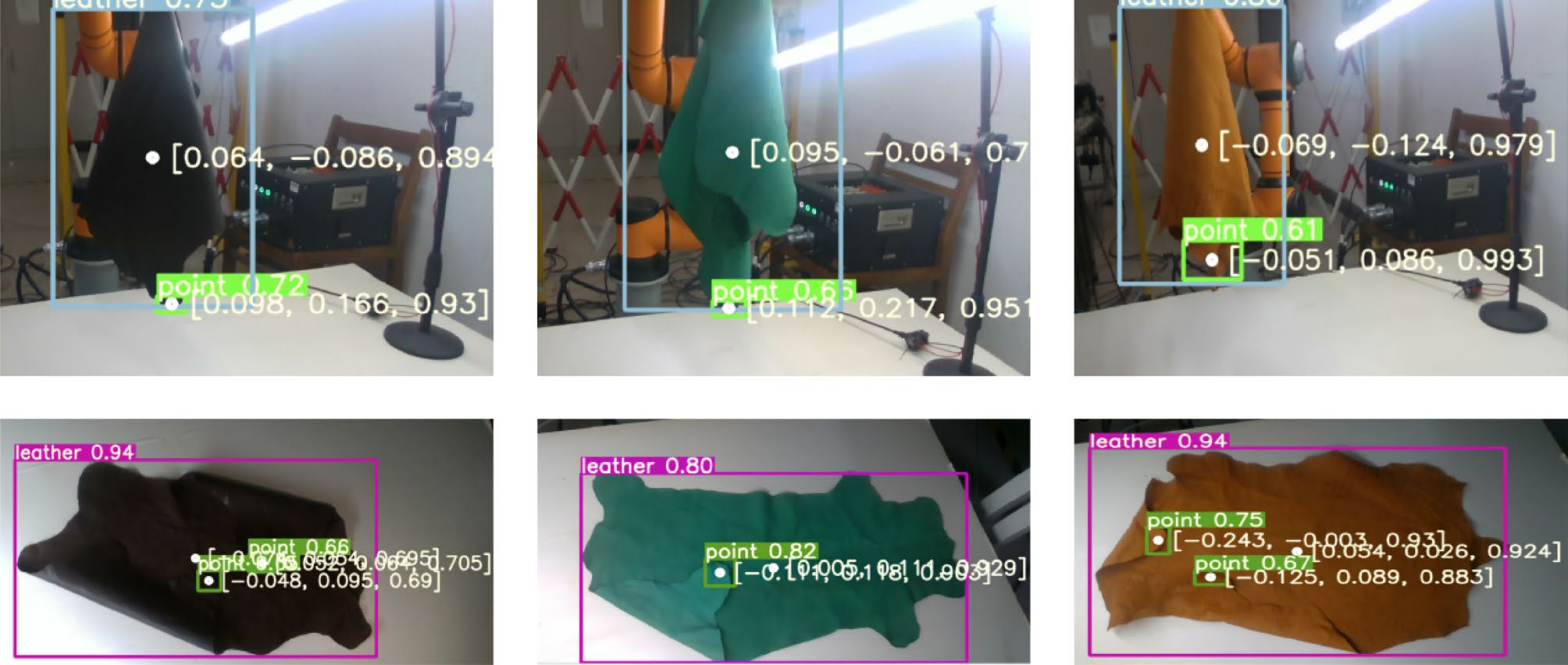
Leather grasping point identification results.

To demonstrate the effectiveness of the improved YOLOV8 algorithm visually, the recognition and localization results for the lowest grasping points and flat spreading grasping points on leather from a non-training datasets are shown in Fig. 7.

The enhancement of the YOLOV8 algorithm is achieved by incorporating the BIFPN network structure and WIOU loss function into the traditional YOLOV8 framework. To validate the effectiveness of each modification, ablation studies are conducted on the improved modules. Four sets of experiments are carried out, testing two datasets separately and comparing their mean Average Precision (mAP). The resultsare shown in Table 2, with the final enhanced algorithm achieving an increase in mAP by 0.75% and 0.41% for the two datasets respectively. which indicates that each step of improvement on the traditional YOLOV8 algorithm is effective.

**Table 2:** Ablation Experiment Results.

Experiment number	BIFPN	WIOU	mAP/% (Dataset 1)	mAP/% (Dataset 2)
1	×	×	98.42	98.64
2	√	×	98.67	98.81
3	×	√	98.69	98.76
4	√	√	**99.17**	**99.05**

Significant values are in bold.

**Figure 8:**
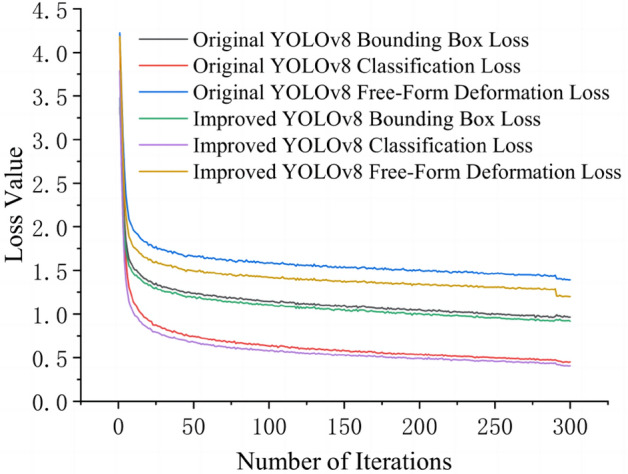
Training loss function.

For a clear visual representation of the performance enhancement in the improved YOLOV8 algorithm, the loss functions of the traditional YOLOV8 and the enhanced YOLOV8 are visualized using the lowest grasping point dataset as an example, which are depicted in Fig. 8. The visualization reveals that the enhanced algorithm exhibits a faster decrease in loss, lower overall loss, and better prediction performance.

## Grasping posture optimization

**Figure 9:**
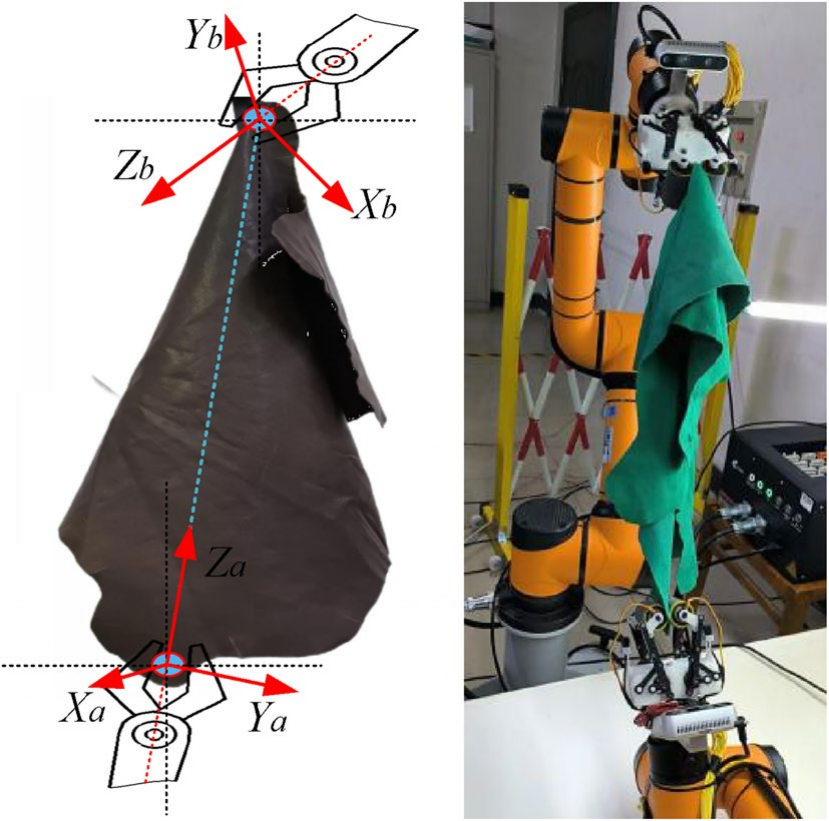
Optimal grasping posture and grasping process diagram.

Initial pre-experiments reveals that controlling the robotic arm to move the end-effector to the identified lowest grasping point directly often leads to failed attempts. The analysis suggests that leather, unlike rigid objects, the grasping success rate is significantly affected by any contact with the leather during the arm's motion or improper grasping postures of the end-effector. The fundamental issue is identified as preventing the leather from swaying due to unsuitable grasping postures during the arm’s movement. Through extensive experimentation, aligning the lower grasping manipulator’s approach with the extension line from the upper manipulator to the identified grasping point mitigates the leather sway caused by the arm’s motion and the manipulator’s grasping posture effectively, which increases the success rate of grasping significantly. Consequently, the optimal grasping posture is determined to be where the lower manipulator aligns with the extension line from the upper manipulator to the identified grasping point. The principle diagram and the grasping process are illustrated in Fig. 9.

## Grasping and spreading experiments

**Figure 10:**
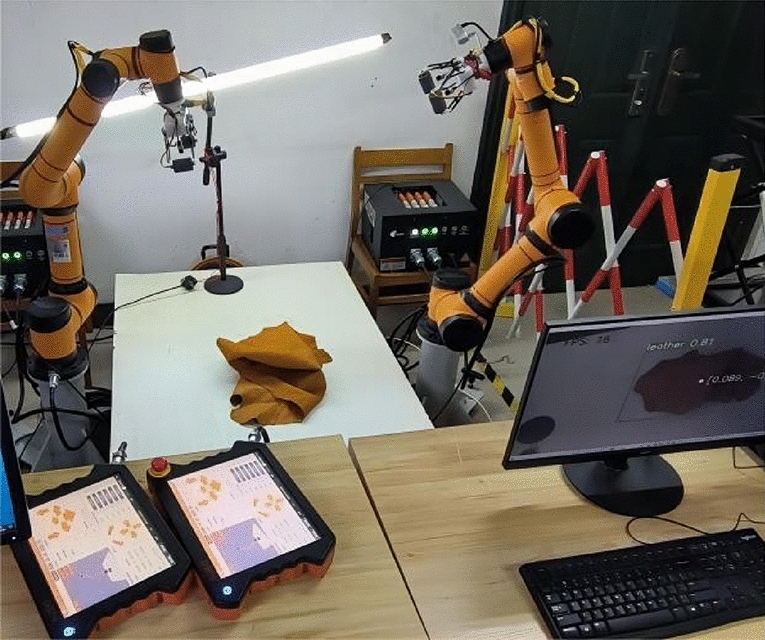
Dual-arm robotic experiment platform.

**Figure 11:**
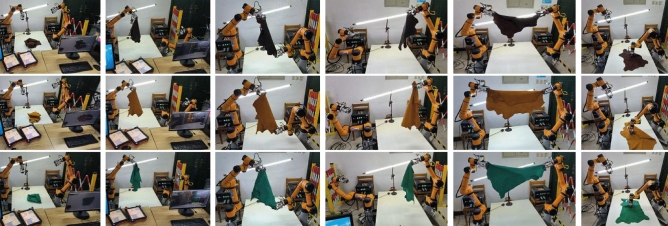
Leather grasping and spreading experimental procedure diagram.

To validate the effectiveness of the dual-arm grasping and spreading strategy and the identification algorithm, an experimental platform is constructed using two AUBO-i5 robotic arms with wheeled end-effectors^24^, Intel RealSense D435i depth camera, light source, a work platform, and a supervisory computer, as shown in Fig. 10. The leather samples used in the experiments had a maximum area of 1200 × 1000 mm. To meet the requirements of dual-arm collaboration and ensure the leather can be fully spread, AUBO-i5 robotic arms with a working radius greater than 850 mm and a minimum load capacity of 3 kg were selected. The two robotic arms were positioned 1200 mm apart, and the depth camera was configured in an eye-in-hand layout. 50 grasping and spreading experiments on leather of different colors and shapes by the platform. The experiments are divided into 5 main steps, with the number of successful operations recorded for each step. An experiment is considered successful entirely if all steps are completed successfully, and the success rate is calculated as the ratio of the total number of successful experiments to the total number of experiments. Part of the experimental process is shown in Fig. 11, and the successful experimental data are recorded in Table 3.

**Table 3:** Experimental Results Record Table.

Experimental steps	A (Left robotic arm)		B (Right robotic arm)		C (Left robotic arm)		D (Dual-arm spreading)		E (Flat grasping)		Total number of Successes	Success rate
	Identification	Grasping	Identification	Grasping	Identification	Grasping	Stretching	Spreading	Identification	Grasping		
Number of valid experiments	50	50	49	46	45	43	41	41	36	36	36	72%
Number of successful attempts	50	49	46	45	43	41	41	36	36	36		
Number of failed attempts	0	1	3	1	2	2	0	5	0	0		

According to the results in Table 3, the overall success rate of the experiment is 72%. Analysis of each experimental step reveals that failures mainly occur during the processes of grasping by the manipulator, dual-arm spreading, and recognition of the lowest point. The initial recognition of the randomly stacked leather is accurate due to the contrasting color difference between the leather and the uniform background of the work platform. However, during the first grasping attempt of the randomly stacked leather by the manipulator unreliable grasping occurs occasionally due to varying grasping positions, which results in the leather falling during the up-and-down shaking motion intended to spread the leather. The recognition of the first lowest grasping point by the right-side camera is sometimes unsuccessful or incorrect due to lighting effects and cluttered background. Failures during the grasping of the lowest point by the right robotic arm occurs occasionally because the arm's movement causes the leather to sway, which leads to a failed grasp. The reasons for failures in the second recognition of the lowest grasping point by the left camera and the subsequent grasping by the left manipulator are similar to those in the previous step, mainly due to incorrect recognition caused by lighting and background, as well as grasping failures due to the arm's movement causing the leather to sway. During the process of both arms stretching the leather to spread out, grasping is reliable and do’nt fail. However, when the arms work together to spread the leather on the table, the inability to determine which side has a smaller folded area sometimes leads to smaller folded areas being covered by larger flat areas during spreading, which prevents subsequent recognition of flat spreading points. If the leather is spread on the table by the collaborative work of both arms successfully, no failures occur in the subsequent recognition and grasping of flat spreading points.

For the entire experimental process, the average time to successfully complete one full experiment was 68 s, with the fastest time being 42 s and the slowest time being 103 s. The relatively long duration of the entire experiment is due to the lack of optimization of the experimental platform’s speed. The most time-consuming step in the entire process is the coordination of the dual robotic arms to spread the leather onto the table, which can be improved by optimizing the control of the dual robotic arms to increase speed. The next most time-consuming step is the identification of the lowest point. This delay is not due to the slowness of the recognition algorithm, but because the leather tends to sway in its naturally hanging state, leading to inaccurate identification and positioning. Accurate identification of the lowest grasping point requires the leather to be completely still. Future optimization of the entire system could further increase operational speed, making it comparable to manual operation.

Analysis of the entire experimental process and the reasons for failures reveals that the main issues are related to the recognition of the lowest point, the grasping process of the manipulator driven by the robotic arm, and the dual-arm spreading process. For the recognition of the lowest point, future research will avoid incorrect recognition effectively by improving lighting conditions and minimizing the impact of cluttered backgrounds. To address the issue of touching the leather during the grasping process which led to failures, future research will take the leather as an obstacle to be avoided by the robotic arm during its movement, prevent the leather from swaying and increase the success rate. Regarding the issue of smaller folded areas being covered by larger flat areas during dual-arm spreading, the current experimental platform is limited in addressing this issue. Future research will introduce two additional third-angle views to determine which side has a larger flat area, allow the arms to spread in that the corresponding direction and avoid experiment failures caused by this issue potentially. A comprehensive analysis of the entire experimental process suggests that further improvement in the success rate requires optimization of multiple operational details, which will be the focus of ongoing research.

## Conclusion

This study delves into the challenges associated with robotic manipulation of irregularly edged sheet-like soft objects and materials, particularly leather. Despite extensive research in this field, developing a practical and efficient method remains a significant challenge. This research proposes an innovative Lowest-Two-Point-Plane (LTPP) method addressing the irregularity of leather edges, surface stains, and the ambiguity of characteristic recognition points, effectively achieve precise grasping and spreading of leather. Moreover, the study provides a detailed explanation of the operational process.

To address the accurate identification of the lowest grasping points on leather, the existing YOLOV8 algorithm is enhanced by incorporating a BiFPN network structure and WIOU loss function. Through training on the lowest and flat spreading grasping points datasets, the accuracy of identification is improved significantly. Additionally, the optimal grasping posture of the robotic arm is determined through pre-experiments.

Through 50 leather grasping and spreading experiments, this research not only validates the effectiveness of the proposed method and algorithm but also explores potential improvements to increase the success rate of operations. The findings confirm the feasibility of using robots to replace manual labor in leather manufacturing processes, Which is of strategic importance for promoting the development of the industry towards intelligence. Future works will focus on further exploring and optimizing this method to achieve precise manipulation of more complex and irregularly shaped sheet-like soft materials, embroad its application sphere.

## Data Availability

The datasets used and/or analysed during the current study available from the corresponding author on reasonable request.

## References

[1] Triantafyllou D, Mariolis I, Kargakos A, Malassiotis S, Aspragathos N (2016). A geometric approach to robotic unfolding of garments. Robot. Auton. Syst..

[2] Osawa F, Seki H, Kamiya Y (2007). Unfolding of massive laundry and classification types by dual manipulator. J. Adv. Comput. Intell. Intell. Inform..

[3] Cusumano-Towner, M., Singh, A., Miller, S., O'Brien, J. F. & Abbeel, P. Bringing clothing into desired configurations with limited perception. In *2011 IEEE International Conference on Robotics and Automation*, 3893–3900 (IEEE, 2011).

[4] Li, Y., Wang, Y., Case, M., Chang, S. F. & Allen, P. K. Real-time pose estimation of deformable objects using a volumetric approach. In *2014 IEEE/RSJ International Conference on Intelligent Robots and Systems*, 1046–1052 (IEEE, 2014).

[5] Kuribayashi, Y., Yoshioka, Y., Onda, K., Yamazaki, T., Wu, T., Arnold, S. & Yamazaki, K. A dual-arm manipulation system for unfolding and folding rectangular cloth. In *2023 IEEE International Conference on Mechatronics and Automation (ICMA)*, 972–978 (IEEE, 2023).

[6] Estevez, D., Fernandez-Fernandez, R., Victores, J. G. & Balaguer, C. Improving and evaluating robotic garment unfolding: A garment-agnostic approach. In *2017 IEEE International Conference on Autonomous Robot Systems and Competitions (ICARSC)*, 210–215 (IEEE, 2017).

[7] Proesmans, R. & Verleysen, A. UnfoldIR: Tactile Robotic Unfolding of Cloth. *IEEE Robot. Autom. Lett.* (2023).

[8] Maitin-Shepard, J., Cusumano-Towner, M., Lei, J. & Abbeel, P. Cloth grasp point detection based on multiple-view geometric cues with application to robotic towel folding. In *ICRA*, 2308–2315 (2010).

[9] Yamazaki, K. Grasping point selection on an item of crumbled clothing based on relational shape description. In *IROS*, 3123–3128, IROS, 3123–3128 (2014).

[10] Yamazaki, K. A method of grasp point selection from an item of clothing using hem element relations. *Adv. Robot.* **29**(1) (2015).

[11] Kita, Y., Ueshiba, T., Neo, E. S. & Kita, N. Clothes state recognition using 3D observed data. In *ICRA*, 480–485 (2009).

[12] Li, Y., Wang, Y., Case, M., Chang, S.-F. & Allen, K. P. Real-time estimation of deformable objects using a volumetric approach. In *IROS*, 987–993 (2014).

[13] Li, Y., Xu, D., Yue, Y., Wang, Y., Chang, S.-F., Grinspun, E. & Allen, P. K., Regrasping and unfolding of garments using predictive thin shell modelling. In *ICRA* (2015).

[14] Doumanoglou, A., Kargakos, A., Kim, T.-K. & Malassiotis, S. Autonomous active recognition and unfolding of clothes using random decision forests and probabilistic planning. In *ICRA*, 987–993 (2014).

[15] Hamajima K, Kakikura M (2000). Planning strategy for task of unfolding clothes. Robot. Auton. Syst..

[16] Hamajima K, Kakikura M (2000). Planning strategy for task of unfolding clothes—classification of clothes. J. Robot. Mechatron..

[17] Kaneko, M. & Kakikura, M. Study on handling clothes-task planning of deformation for unfolding laundry. *J. Robot. Mechatron*. **15**(4) (2003).

[18] Osawa F, Seki H, Kamiya Y (2007). Unfolding of massive laundry and classification types. J. Adv. Comp. Intell. Intell. Inf..

[19] Cusumano-Towner, M., Singh, A., Miller, S., O’Brien, J. F. & Abbeel, P. Bringing clothing into desired configurations with limited perception. In *ICRA*, 3893–3900 (2011)

[20] Yang JD, Shang SY (2013). Cloth modeling simulation based on mass spring model[J]. Appl. Mech. Mater..

[21] Dong, M. & Yuan, Y. 3D garment simulation and visualization based on particle spring model[C]. In *2018 3rd Joint International Information Technology, Mechanical and Electronic Engineering Conference (JIMEC 2018)*, 233–237 (Atlantis Press, 2018)

[22] Wang X, Gao H, Jia Z (2023). BL-YOLOv8: An improved road defect detection model based on YOLOv8[J]. Sensors.

[23] Aboah, A., Wang, B., Bagci, U. *et al*. Real-time multi-class helmet violation detection using few-shot data sampling technique and yolov8[C]. In *Proceedings of the IEEE/CVF Conference on Computer Vision and Pattern Recognition*, 5349–5357 (2023).

[24] Huan Y, Ren G, Su X (2023). A versatile end effector for grabbing and spreading of flaky deformable object manipulation[J]. Mech. Sci..

